# Activation of PERK/eIF2α/ATF4/CHOP branch of endoplasmic reticulum stress response and cooperation between HIF-1α and ATF4 promotes Daprodustat-induced vascular calcification

**DOI:** 10.3389/fphar.2024.1399248

**Published:** 2024-07-31

**Authors:** Andrea Tóth, Gréta Lente, Dávid Máté Csiki, Enikő Balogh, Árpád Szöőr, Béla Nagy, Viktória Jeney

**Affiliations:** ^1^ MTA-DE Lendület Vascular Pathophysiology Research Group, Research Centre for Molecular Medicine, Faculty of Medicine, University of Debrecen, Debrecen, Hungary; ^2^ Doctoral School of Molecular Cell and Immune Biology, Faculty of Medicine, University of Debrecen, Debrecen, Hungary; ^3^ Department of Biophysics and Cell Biology, Faculty of Medicine, University of Debrecen, Debrecen, Hungary; ^4^ Department of Laboratory Medicine, Faculty of Medicine, University of Debrecen, Debrecen, Hungary

**Keywords:** chronic kidney disease (CKD), vascular calcification, prolyl hydroxylase inhibitor, hypoxia-inducible factor 1, endoplasmic reticulum stress, ATF4, Daprodustat

## Abstract

**Introduction:** Vascular calcification is accelerated in patients with chronic kidney disease (CKD) and increases the risk of cardiovascular events. CKD is frequently associated with anemia. Daprodustat (DPD) is a prolyl hydroxylase inhibitor for the treatment of CKD-associated anemia that enhances erythropoiesis through the activation of the hypoxia-inducible factor 1 (HIF-1) pathway. Studies showed that DPD promotes osteogenic differentiation of human aortic smooth muscle cells (HAoSMCs) and increases aorta calcification in mice with CKD. HIF-1 activation has been linked with endoplasmic reticulum (ER) stress; therefore, here we investigated the potential contribution of ER stress, particularly activating transcription factor 4 (ATF4), to the pro-calcification effect of DPD.

**Methods:** Here, we used an adenine-induced CKD mouse model and HAoSMCs as an *in vitro* vascular calcification model to study the effect of DPD.

**Results:** DPD treatment (15 mg/kg/day) corrects anemia but increases the expression of hypoxia (Glut1, VEGFA), ER stress (ATF4, CHOP, and GRP78), and osteo-/chondrogenic (Runx2, Sox9, BMP2, and Msx2) markers and accelerates aorta and kidney calcification in CKD mice. DPD activates the PERK/eIF2α/ATF4/CHOP pathway and promotes high phosphate-induced osteo-/chondrogenic differentiation of HAoSMCs. Inhibition of ER stress with 4-PBA or silencing of ATF4 attenuates HAoSMC calcification. DPD-induced ATF4 expression is abolished in the absence of HIF-1α; however, knockdown of ATF4 does not affect HIF-1α expression.

**Conclusion:** We concluded that DPD induces ER stress *in vitro* and *in vivo*, in which ATF4 serves as a downstream effector of HIF-1 activation. Targeting ATF4 could be a potential therapeutic approach to attenuate the pro-calcific effect of DPD.

## 1 Introduction

CKD is frequently associated with cardiovascular calcification, mainly driven by hyperphosphatemia, a well-characterized calcification inducer (Giachelli, 2009; Ogata et al., 2024). CKD-associated calcification participates in disease progression and the development of cardiovascular complications, which are the major causes of death in CKD patients (Mizobuchi et al., 2009; Zoccali et al., 2023).

Anemia is common and contributes to the increased mortality and morbidity of CKD patients (Hanna et al., 2021; Atkinson and Warady, 2018; Kovesdy et al., 2023). The current standard of anemia treatment is intravenous iron supplementation together with the administration of erythropoiesis-stimulating agents (ESAs) (Hanna et al., 2021). Unfortunately, studies showed that ESAs increase the probability of major cardiovascular events (MACE) in CKD patients (Babitt and Lin, 2012; Portolés et al., 2021). Prolyl hydroxylase domain-containing (PHD) enzyme inhibitors represent a new concept in treating CKD-associated anemia through the activation of the hypoxia-inducible factor (HIF) pathway and subsequent erythropoiesis (Mima, 2021).

Numerous clinical trials have been completed with three different PHD inhibitors Roxadustat, Vadadustat, and Daprodustat (DPD) concluding that these orally administrable compounds are effective and safe alternatives to ESAs for anemia treatment in CKD patients. All of the compounds are approved for marketing in Japan, Roxadustat is approved in China and DPD is the only one approved by the United States Food and Drug Administration (FDA) for anemia management in CKD patients. On the other hand, according to the ASCEND-D trial, DPD is not a safer alternative in comparison to ESAs for the occurrence of MACE in CKD patients (Singh et al., 2021). Previously, we showed that DPD promotes CKD-associated vascular and aortic valve calcification via the activation of the HIF pathway (Tóth et al., 2022; Csiki et al., 2023). However, the involvement of other molecular mechanisms by which DPD could contribute to MACE in CKD patients remained unclear.

The endoplasmic reticulum (ER) is a multifunctional organelle that plays important roles in protein folding, assembly, secretion, lipid synthesis, and calcium homeostasis (Lin et al., 2008; Walter and Ron, 2011). Various types of stress, e.g., starvation, hypoxia, certain drugs, toxins, etc., can trigger disruption of ER homeostasis (Lin et al., 2008; Walter and Ron, 2011). Cells respond to ER stress by activating a complex signal transduction pathway known as the unfolded protein response (UPR) through three stress sensor proteins, i.e., protein kinase RNA-like ER kinase (PERK), inositol-requiring protein 1α (IRE1α), and activating transcription factor 6 (ATF6) (Ron and Walter, 2007; Hetz, 2012). UPR can trigger adaptive responses, or if ER stress is sustained, it can lead to apoptosis. PERK phosphorylates the alpha subunit of eukaryotic initiation factor 2 (eIF2α), leading to a nearly global translational arrest and selective translation of activating transcription factor 4 (ATF4). Transcriptional factor C/EBP homologous protein (CHOP) is an important target of ATF4, which promotes ER stress-induced apoptosis when restoration of ER homeostasis fails (Ron and Walter, 2007; Hetz, 2012). ATF4 is an essential transcription factor that mediates not only ER stress but also the terminal differentiation of osteoblasts by regulating osteoblast-specific gene expressions (Yang et al., 2004; Karsenty, 2008). Additionally, ATF4 actively participates in the phenotype switch of vascular smooth muscle cells (VSMCs) into osteoblast-like cells and subsequent vascular calcification, which notion is supported by the attenuation of CKD-driven aortic calcification in vascular smooth muscle cell-specific ATF4-deficient mice (Masuda et al., 2016; Rao et al., 2022).

It has been shown that ATF4 is translationally induced by hypoxia and the PHD inhibitor dimethyloxalylglycine (Köditz et al., 2007). We previously reported that DPD accelerates high phosphate-induced calcification of human aortic smooth muscle cells (HAoSMCs) and valve interstitial cells that causes an increase in aortic and valve calcification respectively, in mice with adenine-induced CKD (Tóth et al., 2022; Csiki et al., 2023).

Thus, we postulated that DPD-induced vascular calcification involves the activation of ER stress. In this study, we investigated whether 1) DPD induces ER stress and hypoxia in adenine-induced CKD mice, 2) DPD upregulates osteogenic markers and promotes calcification in adenine-induced CKD mice, 3) DPD induces PERK phosphorylation, ATF4, glucose-regulated protein 78 (GRP78), and CHOP expression in HAoSMCs, 4) DPD promotes calcification and osteogenic differentiation of HAoSMCs in an ER-stress and ATF4-dependent manner, and 5) there is a hierarchy between DPD-induced HIF-1α and ATF4 responses.

## 2 Materials and methods

### 2.1 Materials

The detailed list of materials (company name, catalog number, sequences, etc.) can be found in the “Resources table” in the Supplementary Material.

### 2.2 Cell culture and treatments

HAoSMCs were maintained in Dulbecco’s Modified Eagle Medium (DMEM) supplemented with 10% fetal bovine serum (FBS), antibiotic antimycotic solution, sodium pyruvate, and L-glutamine. Cells were maintained at 37°C in a humidified atmosphere with 5% CO2. Cells were grown to ∼90% confluency and used between passages five and 8. To induce calcification, HAoSMCs were exposed to an osteogenic medium (OM) that was obtained by supplementing the growth medium with inorganic phosphate (Pi) (NaH_2_PO_4_-Na_2_HPO_4_, 1–2.5 mmol/L, pH 7.4). DPD was utilized at concentrations ranging from 1 to 100 μmol/L after being dissolved in dimethyl sulfoxide (DMSO) to create a stock solution (25 mmol/L). In some experiments, we used sodium-4-phenylbutyrate (4-PBA, stock solution: 50 mmol/L in DMSO, working concentration: 250 μmol/L) to inhibit ER stress.

### 2.3 Alizarin red (AR) staining and quantification

After washing with Dulbecco’s phosphate buffered saline (DPBS), the cells were fixed in 4% paraformaldehyde for 20 min and rinsed with distilled water. Cells were stained with Alizarin Red S solution (2%, pH 4.2) for 10 min at room temperature. Excessive dye was removed by several washes in distilled water. To quantify AR staining, we added 100 μL of hexadecyl-pyridinium chloride solution (100 mmol/L) to each well and measured optical density (OD), using a microplate reader at 560 nm.

### 2.4 Quantification of Ca deposition

Cells grown on 96-well plates were washed twice with DPBS and decalcified with HCl (0.6 mol/L) for 30 min. The Ca content of the HCl supernatants was determined by the QuantiChrome Calcium Assay Kit. Following decalcification, cells were washed with DPBS and solubilized with a solution of NaOH (0.1 mol/L) and sodium dodecyl sulfate (0.1%), and the protein content of the samples was measured with the BCA protein assay kit. The Ca content of the cells was normalized to protein content and expressed as μg/mg protein.

### 2.5 Osteocalcin (OCN) detection

Cells grown on 6-well plates were washed twice with DPBS and decalcified with 100 μL of EDTA (0.5 mol/L, pH 6.9) for 30 min. OCN content of the EDTA-solubilized ECM samples was quantified by an enzyme-linked immunosorbent assay according to the manufacturer’s protocol.

### 2.6 *Ex vivo* aorta organ culture model and quantification of aortic ca

C57BL/6 mice (8–12-week-old male, n = 18) were exterminated by CO_2_ inhalation and perfused with 5 mL of sterile DPBS. The entire aorta was harvested and cleaned under aseptic conditions, and cut into pieces. Aorta rings were maintained in control, high Pi + DPD (25 μmol/L), and high Pi+4-PBA (250 μmol/L) in DMEM supplemented with 10% FBS, antibiotic antimycotic solution, sodium pyruvate, L-glutamine, and 2.5 μg/mL Fungizone. After 7 days, the aorta pieces were washed in phosphate-balanced saline (PBS), opened longitudinally, and decalcified in 25 µL of 0.6 mmol/L HCl overnight. Ca content was determined by the QuantiChrom Ca-assay kit, as described previously.

### 2.7 CKD induction, DPD treatment and near-infrared imaging and quantification of aortic calcification in mice

Animal care and experimental procedures were performed following the institutional and national guidelines and were approved by the Institutional Ethics Committee of the University of Debrecen under registration number 10/2021/DEMÁB. Animal studies were reported in compliance with the ARRIVE guidelines. All the mice were housed in a temperature- (22°C) and light-controlled (12-h light/12-h dark) room, in cages with standard beddings and unlimited access to food and water. C57BL/6 mice (10 weeks old, male, n = 30) were randomly divided into three groups: control (Ctrl), CKD, and CKD + DPD (CKDD) (10 mice/group). CKD was induced by a two-phase diet, as described previously (Tani et al., 2017). In the first 6 weeks, the mice received a diet containing 0.2% adenine and 0.7% phosphate, followed by a diet containing 0.2% adenine and 1.8% phosphate for 3 weeks. Ctrl mice received a normal chow diet. DPD was suspended in 1% methylcellulose and administered orally at a dose of 15 mg/kg/day from week 7. Following the 9-week diet five mice/group were anesthetized with isoflurane and injected retro-orbitally with 2 nmol of OsteoSense dye that was dissolved in 100 µL of PBS. Twenty-4 hours later, mice were euthanized by CO_2_ inhalation and blood was taken by heart puncture into K3-EDTA-containing tubes. Then mice were perfused with 5 mL of ice-cold PBS. Kidneys and aortas were isolated and analyzed immediately *ex vivo* by an IVIS Spectrum *In Vivo* Imaging System. We took kidney and aorta tissues out from the remaining 15 mice (5 mice/group), snap freeze them in liquid nitrogen and kept at −80°C for further analysis.

### 2.8 Laboratory analysis of renal function and anemia in CKD mice

Serum urea, creatinine, phosphate and calcium levels were determined in mice by kinetic assays on a Cobas^®^ c501 instrument. K3-EDTA anticoagulated whole blood murine samples were analyzed by a Siemens Advia-2120i hematology analyzer with the 800 Mouse C57BL program of Multi-Species software. Hemoglobin concentration was measured by a cyanide-free colorimetric method. Hematocrit values were determined as a calculated parameter derived from red blood cell count (RBC in T/L) and mean cell volume (MCV in fL).

### 2.9 Real-time quantitative polymerase chain reaction (qPCR)

Total RNA was extracted from the kidney and aorta of C57BL/6 mice using Tri Reagent following the manufacturer’s protocol. RNA was reverse transcribed using a High-Capacity cDNA Reverse Transcription Kit. The qPCR reactions were carried out according to the protocol of the iTaq universal SYBR^®^ Green Supermix reagent, using primers listed in the “Resources table.” PCR was performed using a real-time PCR machine.

### 2.10 Western blot analysis

HAoSMCs were lysed in Laemmli lysis buffer. Proteins were resolved by SDS-PAGE (7.5% and 10%) and transferred onto nitrocellulose membranes. Western blotting was performed with the use of the primary antibodies listed in the “Resources table.” Following the primary antibody binding, membranes were incubated with horseradish peroxidase-linked rabbit and mouse IgG. Antigen-antibody complexes were visualized with the enhanced chemiluminescence system Clarity Western ECL. Chemiluminescent signals were detected conventionally on an X-ray film or digitally with the use of a C-Digit Blot Scanner. After detection, the membranes were stripped and reprobed for β-actin. Blots were quantified by using the built-in software on the C-Digit Blot Scanner.

### 2.11 RNA silencing

To knockdown ATF4 gene expressions, we used Silencer^®^ select siRNA constructs targeting HIF-1α and ATF4. As a control, we used the negative control #1 construct. Lipofectamine^®^ RNAiMAX reagent was used to transfect HAoSMCs according to the manufacturer’s protocol.

### 2.12 Statistical analysis

Results are expressed as mean ± SD. At least three independent experiments were performed for all *in vitro* studies. Statistical analyses were performed with GraphPad Prism 8.0.1 software. Comparisons between more than two groups were carried out by a one-way ANOVA followed by Tukey’s multiple-comparisons test. To compare each of several treatment groups with a single control group, we performed a one-way ANOVA followed by Dunnett’s *post hoc* test. A value of *p* < 0.05 was considered significant.

## 3 Results

### 3.1 DPD promotes HIF pathway activation and ER stress in the kidney and aorta of CKD mice

**FIGURE 1:**
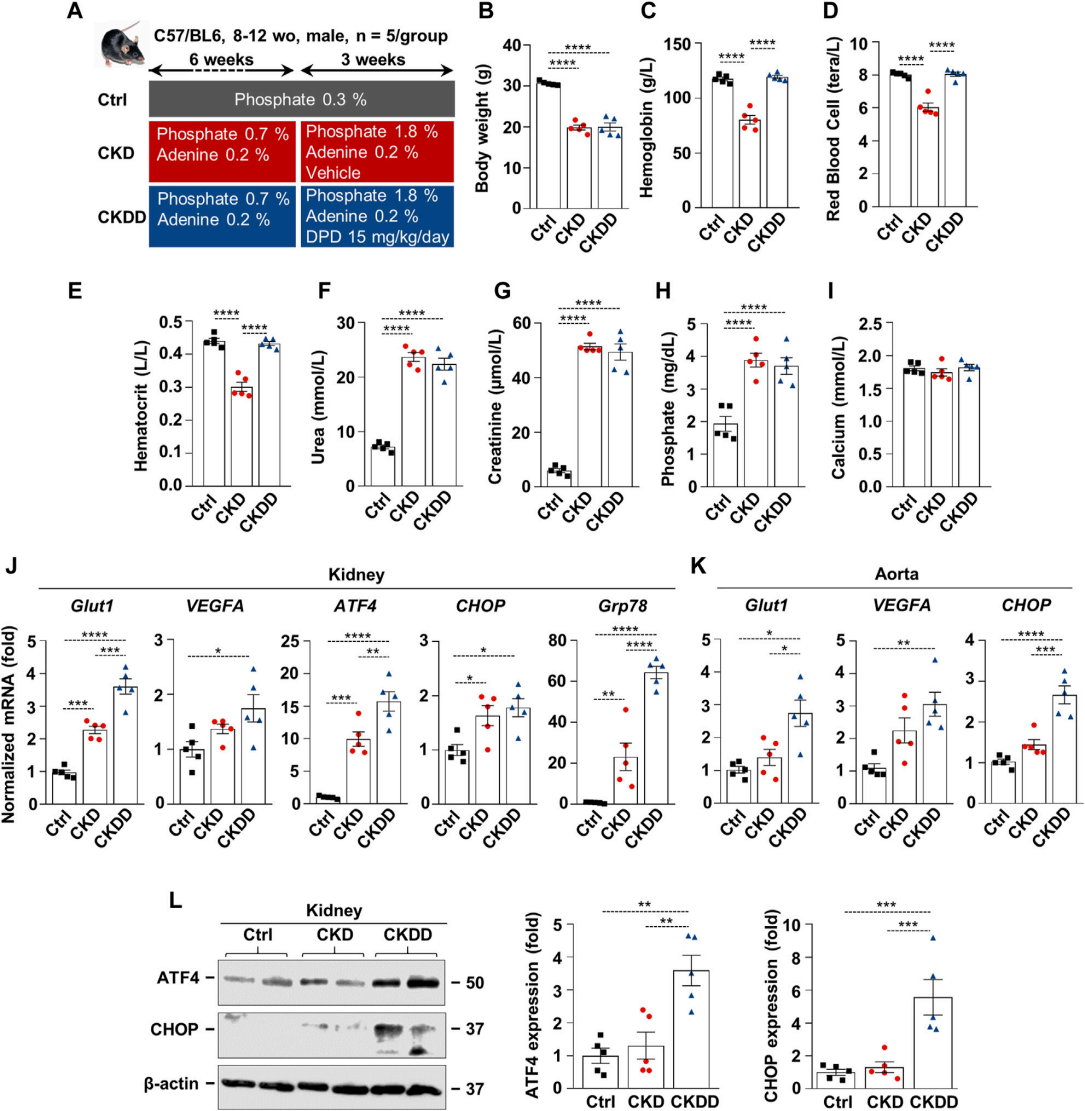
DPD promotes HIF pathway activation and ER stress in kidney and aorta in C57BL/6 mice fed with an adenine + high Pi diet. **(A)** Scheme of the experimental protocol. **(B)** Body weight; **(C)** hemoglobin; **(D)** red blood cell count; **(E)** hematocrit; **(F)** plasma urea; **(G)** creatinine; **(H)** phosphate; and **(I)** calcium level. **(J and K)** mRNA levels of hypoxia and ER stress markers in **(J)** kidney and **(K)** aorta samples. **(L)** Protein expressions of ATF4 and CHOP in kidney lysates. Membranes were reprobed for β-actin. Representative Western blots and analyses (n = 3). Data are expressed as mean ± SD, n = 5. Ordinary one-way ANOVA followed by Tukey’s multiple comparison test was used to calculate *p* values. *p < 0.05, **p < 0.01, ***p < 0.005, ****p < 0.001.p

Fifteen C57BL/6 mice (8–12 weeks old, male) were randomized into three groups (n = 5/group): control (Ctrl), CKD, and CKD treated with DPD (CKDD). CKD was induced with a 9-week-long, two-phase adenine- and high-phosphate-containing diet, as detailed in Figure 1A. DPD was administered orally at a dose of 15 mg/kg/day in the last 3 weeks of the experiment (Figure 1A). Ctrl mice received a normal chow diet. Hematological parameters, body weight, and kidney function were evaluated at the end of the experiment. Both CKD and CKDD mice lost approximately one-third of their initial weight during the experiment (Figure 1B). DPD completely corrected CKD-induced anemia revealed by similar hemoglobin, RBC count, and hematocrit values in Ctrl and CKDD mice (Figures 1C,E). Elevated plasma urea, creatinine, and phosphate levels indicated that the kidney function of the CKDD mice had declined to the same degree as that of the CKD mice (Figures 1F–I). CKD treatment did not change plasma calcium levels (Figure 1I).

CKD was associated with increased renal mRNA expression of specific hypoxia and ER stress markers, such as glucose transporter 1 (Glut1), ATF4, CHOP, and glucose-regulated protein 78 (GRP78) (Figure 1J). DPD treatment further exacerbated CKD-induced activation of HIF-1 target genes and ER stress markers in the kidneys (Figure 1J). In comparison to Ctrl, CKDD treatment triggered a 3-fold increase in Glut1, vascular endothelial growth factor A (VEGFA), and CHOP mRNA expressions in the aorta (Figure 1K). We observed marked upregulation of the protein expressions of ER stress markers ATF4 and CHOP in the kidneys of CKDD mice (Figure 1L).

### 3.2 DPD upregulates markers of osteo-/chondrogenic differentiation and increases kidney and aorta calcification in CKD mice

**FIGURE 2:**
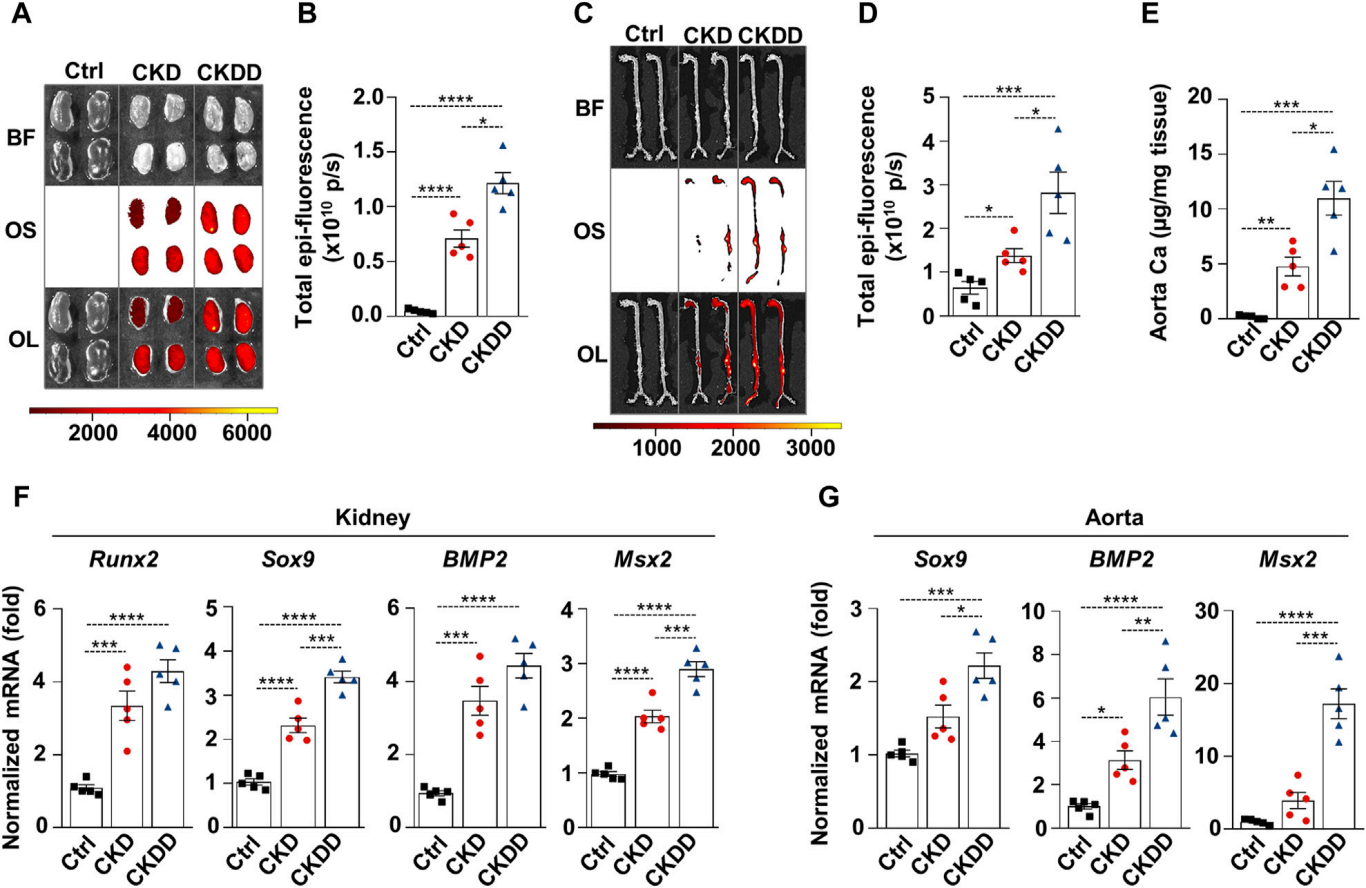
DPD increases soft tissue calcification in the kidney and aorta of CKD mice. Mice were treated as shown in Figure 1A. **(A–D)** Brightfield and macroscopic fluorescence reflectance imaging of **(A)** kidney and **(C)** aorta; **(B and D)** quantification of OsteosenseTM staining. **(E)** Ca content of aortas normalized to protein level. **(F)** mRNA levels of osteogenic markers in kidney and **(G)** aorta samples. Data are expressed as mean ± SD, n = 5. Ordinary one-way ANOVA followed by Tukey’s multiple comparison test was used to calculate *p* values. *p < 0.05, **p < 0.01, ***p < 0.005, ****p < 0.001.

Osteosense staining was performed to evaluate soft tissue calcification in Ctrl, CKD, and CKDD mice. CKD was associated with increased kidney and aorta calcification, which was further exacerbated by DPD treatment (Figures 2A–D). Aorta calcium measurement supported the pro-calcifying effect of DPD in CKD animals (Figure 2E). Calcification is a highly regulated process, similar to bone formation; therefore, next, we investigated the expression of osteo-/chondrogenic markers in kidney and aorta samples. Compared to Ctrl, Runt-related transcription factor 2 (Runx2), SRY-box transcription factor 9 (Sox9), bone morphogenetic protein 2 (BMP2), and Msh Homeobox 2 (Msx2) mRNA levels were higher in the kidneys of CKD mice. Furthermore, we noticed that CKDD mice had higher Sox9 and Msx2 mRNA levels than CKD animals had (Figure 2F). In the aorta, CKD triggered an increase in BMP2 mRNA expression compared to Ctrl, whereas CKDD induced marked elevations of Sox9, BMP2, and Msx2 mRNA levels (Figure 2G). Overall, these results show that DPD treatment induces hypoxia response and ER stress, increases osteo-/chondrogenic marker expressions, and promotes hydroxyapatite deposition in the kidney and aorta of CKD mice.

### 3.3 DPD induces HIF-1 activation and the PERK-eIF2α-ATF4 pathway and promotes high Pi-induced calcification in HAoSMCs

**FIGURE 3:**
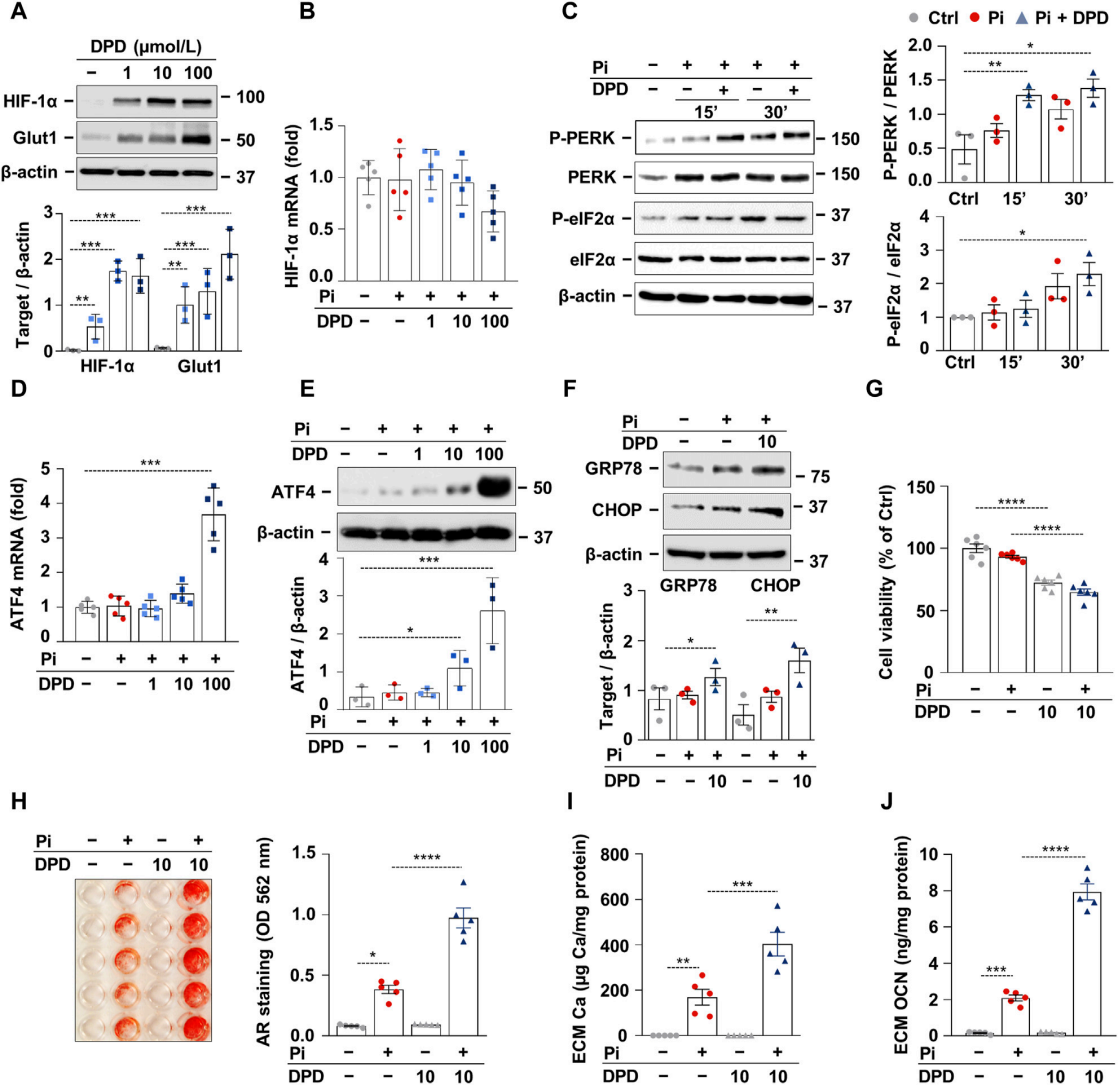
DPD induces hypoxia signaling and endoplasmic reticulum stress and promotes Pi-induced calcification of HAoSMCs. **(A, B)** HAoSMCs were cultured in the presence of DPD (1–100 μmol/L). **(A)** Protein expression of HIF-1α and Glut1 in whole cell lysates was evaluated after 24 h of treatment. Membranes were reprobed for β-actin. Representative Western blots and densitometry analyses on the relative expression of HIF-1α and Glut1 (n = 3). **(B)** mRNA level of HIF-1α after 12 h of treatment. **(C)** HAoSMCs were cultured in the presence or absence of Pi (2 mmol/L) and DPD (10 μmol/L). Protein expression of phospho-PERK (P-PERK), PERK, phospho-eIF2α (P-eIF2α), and eIF2α was measured in whole cell lysates (15 min, 30 min). Membranes were reprobed for β-actin. Representative Western blots and relative expression of P-PERK normalized to PERK and P-eIF2α normalized to eIF2α (n = 3). **(D–F)** HAoSMCs were cultured in the presence of DPD (1–100 μmol/L). **(D)** ATF4 mRNA and **(E–F)** protein expression of ATF4, CHOP, and GRP78 in whole cell lysates (6 h). Membranes were reprobed for β-actin. Representative Western blots and densitometry analyses on the relative expression of ATF4, CHOP, and GRP78 (n = 3). **(H–J)** HAoSMCs were cultured in an osteogenic medium supplemented with phosphate (2 mmol/L Pi) in the presence or absence of DPD (10 μmol/L). **(H)** Representative Alizarin Red staining (day 4) and quantification (n = 5). **(I)** Ca content of HCl-solubilized ECM samples. **(J)** OCN level in EDTA-solubilized ECM samples (day 8). Data are expressed as mean ± SD. Ordinary one-way ANOVA followed by Tukey’s multiple comparison test was used to calculate *p* values. *p < 0.05, **p < 0.01, ***p < 0.005, ****p < 0.001.p

The stress signal network between hypoxia and ER stress is implicated in the progression of CKD; therefore, we further examined the effect of DPD on these pathways using an *in vitro* calcification model. Exposition of HAoSMCs to DPD (1–100 μmol/L) induced stabilization of HIF-1α and subsequent activation of the HIF-1 pathway, as revealed by a dose-dependent increase in Glut-1 protein expression (Figure 3A). We could not detect changes in HIF-1α mRNA levels in DPD-treated HAoSMCs, suggesting that DPD regulates HIF-1α in a post-transcriptional manner (Figure 3B). Hypoxia is a pathophysiological condition that induces ER stress through PERK; therefore, next, we investigated PERK activation in HAoSMCs in response to high Pi (2.5 mmol/L) with or without DPD (10 μmol/L). Pi-induced PERK phosphorylation was further exacerbated by DPD (Figure 3C). Furthermore, compared to control, the levels of phosphorylated eIF2α (P-eIF2α) were elevated by Pi and Pi + DPD (Figure 3C). The activation of the PERK pathway by Pi + DPD induced a massive upregulation of ATF4 mRNA and protein expressions (Figures 3D,E) as well as CHOP, and Grp78 (Figure 3F). Sustained ER stress can induce apoptosis, therefore next we investigated whether DPD influences cell viability. We performed MTT assay, and found that DPD (10 μmol/L) decreased cell viability in both normal and high Pi conditions (Figure 3G). Then, we addressed the pro-calcifying effect of DPD in HAoSMCs. As revealed by Alizarin red staining, DPD (10 μmol/L) largely intensified Pi-induced calcification (Figure 3H). The ECM of HAoSMCs treated with Pi + DPD had approximately 2.4 times more calcium deposition than the ECM of Pi-treated cells (Figure 3I). Moreover, OCN accumulation in the ECM of Pi + DPD-treated HAoSMCs was about 4-times higher compared to Pi-treated cells (Figure 3J).

### 3.4 The pro-calcification effect of DPD is dependent on ER stress activation and ATF4

**FIGURE 4:**
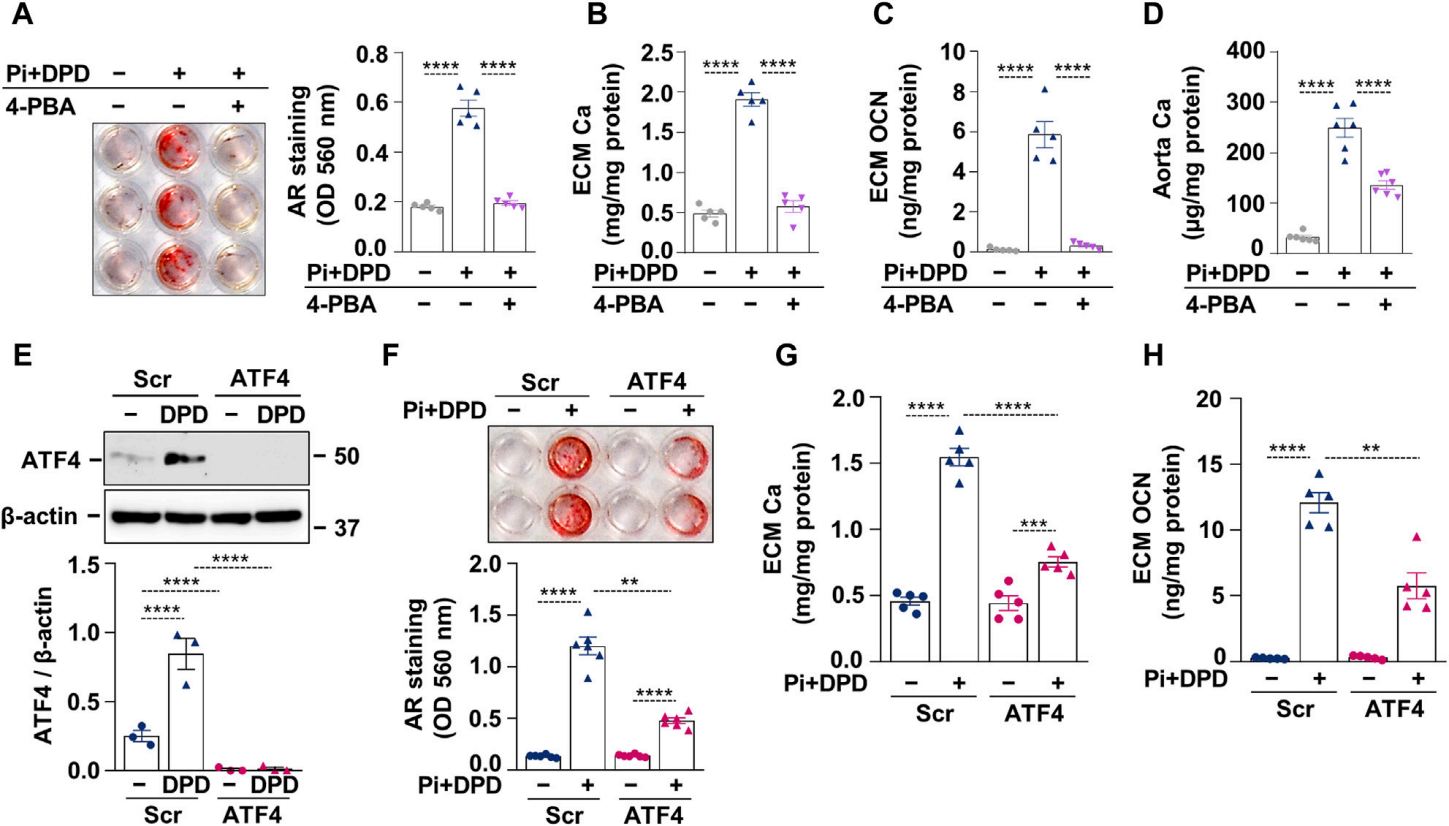
DPD increases the calcification of HAoSMCs through ER stress and ATF4 activation. **(A–C)** HAoSMCs were exposed to high Pi (2 mmol/L) and DPD (10 μmol/L) in the presence or absence of 4-PBA (250 μmol/L). **(A)** Representative AR staining (day 4) and quantification. **(B)** Ca content of HCl-solubilized ECM (day 4). **(C)** OCN level in EDTA-solubilized ECM samples (day 10). **(D)** Aortic rings of C57BL/6 mice were cultured in control, high Pi + DPD (25 μmol/L), and high Pi + DPD+4-PBA conditions. Ca content of aorta rings normalized to protein level (day 7). **(E–H)** HAoSMCs were exposed to Pi (2 mmol/L) and DPD (10 μmol/L) in the presence of ATF4 or scrambled siRNA. **(E)** Protein expression of ATF4 in whole cell lysates (6 h). Membranes were reprobed for β-actin. Representative Western blots and relative expression of ATF4 normalized to β-actin. **(F)** Representative AR staining (day 4) and quantification. **(G)** Ca content of HCl-solubilized ECM (day 4). **(H)** OCN level in EDTA-solubilized ECM samples (day 8). Data are expressed as mean ± SD, n = 3–6. Ordinary one-way ANOVA followed by Tukey’s multiple comparison test was used to calculate *p* values. **p < 0.01, ***p < 0.005, ****p < 0.001.

After establishing that DPD induces ER stress and accelerates high Pi-induced calcification, we investigated whether ER stress plays a causative role in HAoSMC calcification triggered by Pi + DPD. First, we tested the effect of an ER stress inhibitor, 4-phenylbutyrate (4-PBA), on HAoSMC calcification. AR staining revealed that 4-PBA inhibited Pi + DPD-induced calcification of HAoSMCs (Figure 4A). Additionally, 4-PBA inhibited the accumulation of Ca and OCN in the ECM of Pi + DPD-treated HAoSMCs and attenuated *ex vivo* aorta calcification (Figures 4B–D). Furthermore, the knockdown of ATF4 by siRNA decreased Pi + DPD-induced calcification of HAoSMCs as evaluated by AR staining, as well as Ca and OCN measurements from the ECM (Figures 4E–H). These results show that DPD induces ER stress, particularly ATF4, which plays a crucial role in Pi + DPD-induced HAoSMC calcification.

### 3.5 HIF-1α is required for DPD-induced upregulation of ATF4

**FIGURE 5:**
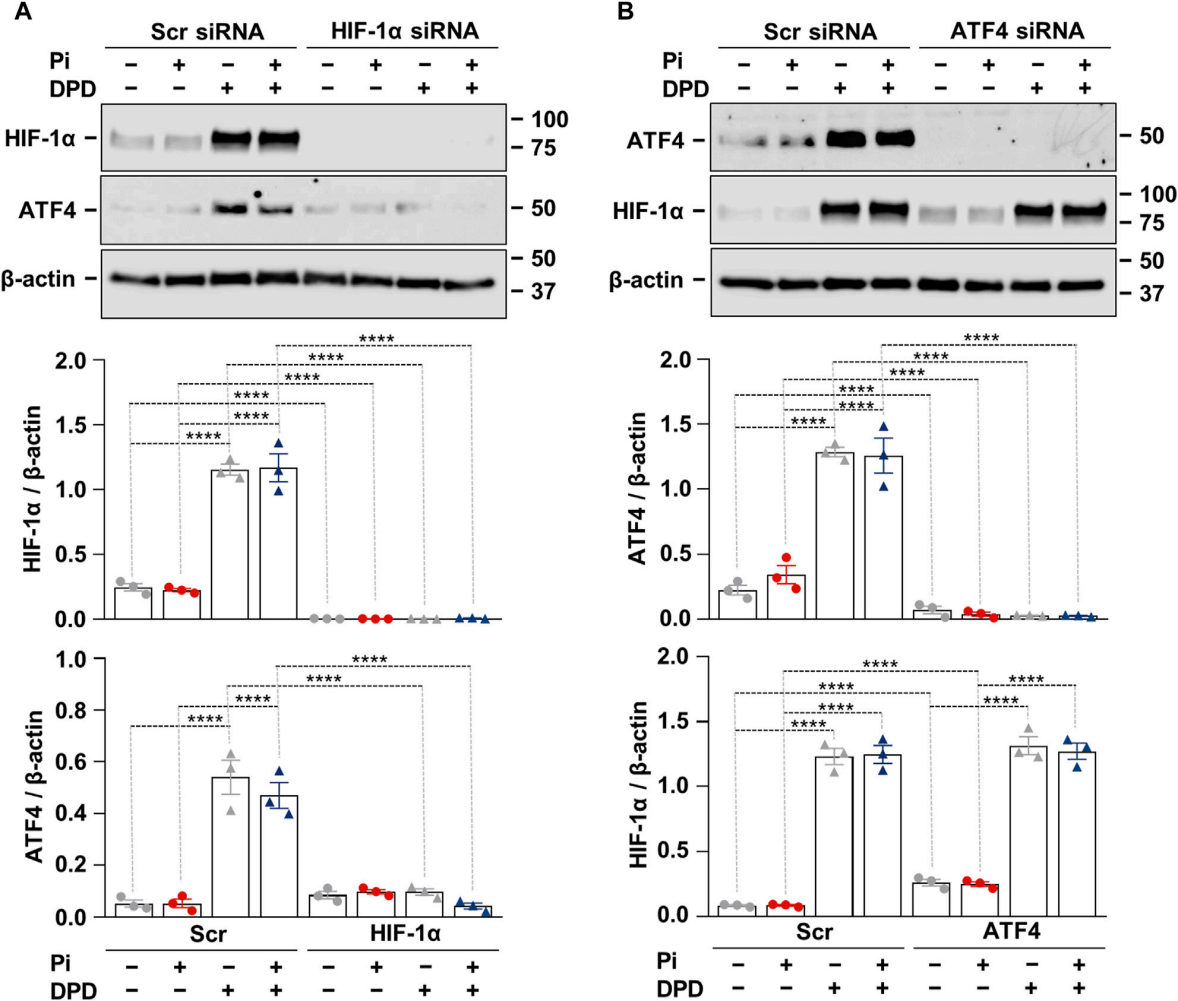
Crosstalk between hypoxia signaling and ER stress in Pi + DPD-induced HIF-1α and ATF4 responses in HAoSMCs. **(A,B)** Cells were exposed to Pi (2 mmol/L) and DPD (10 μmol/L) in the presence of HIF-1α, ATF4, and scrambled siRNA. Protein expression of HIF-1α and ATF4 in whole cell lysates (24 h). Membranes were reprobed for β-actin. Representative Western blots and relative expression of HIF-1α and ATF4 normalized to β-actin. Data are expressed as mean ± SD, n = 3. Ordinary one-way ANOVA followed by Tukey’s multiple comparison test was used to calculate *p* values. **p < 0.01, ****p < 0.001.

After showing that both the HIF-1 pathway and ATF4 activation play essential roles in Pi + DPD-induced HAoSMC calcification, we wanted to understand whether there is a cross-communication between these two pathways. To this end, we applied HIF-1α targeted siRNA and examined the protein expression of HIF-1α and ATF4 in response to Pi (2.5 mmol/L), DPD (10 μmol/L), and Pi + DPD (Figure 5A). Western blot results revealed that the HIF-1α knock-down approach was successful and that in the absence of HIF-1α, DPD fails to upregulate ATF4 expression (Figure 5A). On the other hand, DPD induced HIF-1α expression regardless of the presence of ATF4 (Figure 5B). These results suggest a hierarchy between HIF-1α and ATF4 upon DPD treatment, in which HIF-1α is upstream of ATF4.

## 4 Discussion

The pathomechanism of vascular calcification in CKD is extremely complex and influenced by many factors and molecular pathways (Tóth et al., 2020). Growing evidence suggests that ER stress is a major contributor to vascular calcification (Duan et al., 2009; Liberman et al., 2011; Masuda et al., 2012; Masuda et al., 2013; Shanahan and Furmanik, 2017; Furmanik et al., 2021). In the present study, we found that DPD promotes vascular calcification through the coordinated activation of the HIF-1 pathway and the PERK–eIF2α–ATF4–CHOP axis.

The first important observation of this study is that DPD increases HIF activation, generates ER stress, and promotes kidney and aorta calcification in CKD mice (Figures 1, 2). In this work, we used a non-invasive, well-characterized CKD model in which we induced tubular damage by an adenine-containing diet (Tani et al., 2017). Previously, we showed that these CKD mice are anemic and titrated out the dose of DPD that corrects CKD-induced anemia in this model (Tóth et al., 2022; Csiki et al., 2023). Using the minimal anemia-correcting dose of DPD, we observed an elevation of the mRNA level of the HIF target genes Glut1 and VEGFA in both the kidney and the aorta (Figure 1). This is in agreement with our previous *in vitro* results, in which we showed that PHD inhibitors, including DPD, stabilize HIF-α subunits, activate HIF signaling, and upregulate Glut1 and VEGFA in HAoSMCs and valve interstitial cells (Tóth et al., 2022; Csiki et al., 2023).

A growing body of evidence suggests that hypoxia and ER stress signaling are interconnected and implicated in the pathogenesis of various diseases, including CKD (Maekawa and Inagi, 2017; Díaz-Bulnes et al., 2020). Hypoxia and the PHD inhibitor CoCl_2_ activate PERK and phosphorylate eIF2α in embryonic fibroblasts (Koumenis et al., 2002). It is interesting to note that PHD inhibition attenuates post-ischemic myocardial damage in hearts challenged by ischemia/reperfusion by inducing ER stress proteins including ATF4 and GRP78 while also lowering the level of pro-apoptotic component CHOP (Pereira et al., 2014). The interplay between HIF and ER stress pathways is well-known in tumor biology and serves as an important adaptation mechanism (Lin et al., 2024).

Our results revealed that mRNA levels of ER stress markers (ATF4, CHOP, and GRP78) are elevated in the kidneys of CKD mice, and DPD triggers further increases in these markers. Additionally, we showed that DPD treatment upregulates protein expression of ATF4 and CHOP in the kidneys of CKDD mice (Figure 1). Vascular calcification is a common feature of CKD and contributes to the increased morbidity and mortality of CKD patients. Here we found that HIF activation and ER stress observed in CKDD mice are accompanied by increased kidney and aorta calcification and elevation of mRNA markers of osteo-/chondrogenic differentiation (Runx2, Sox9, BMP2, and Msx2) as compared to CKD mice (Figure 2).

An additional noteworthy finding of this investigation is that DPD stimulates the PERK–eIF2α–ATF4–CHOP axis, hence facilitating high Pi-induced calcification *in vitro* in HAoSMCs (Figure 3). In agreement with our results, previous studies showed that PHD inhibitors are capable of activating the PERK–eIF2α branch of UPR; as such, CoCl_2_ triggers PERK and eIF2α activation in embryonic fibroblasts, and dimethyloxalylglycine stabilizes ATF4 in HeLa cells (Koumenis et al., 2002; Köditz et al., 2007).

Failure of ER stress resolution via UPR may lead to the activation of pro-apoptotic mechanisms. A recent study showed that activation of the PERK-eIF2α-ATF4-CHOP pathway is involved in Arnicolide D-induced oncosis in hepatocellular carcinoma cells (Lin et al., 2024). Here we showed that DPD decreases the viability of HAoSMCs but further investigation is needed to clarify the type of DPD-induced cell death and the potential involvement of the PERK-eIF2α-ATF4-CHOP pathway.

Accumulating evidence suggests the critical involvement of ER stress activation in the transition of smooth muscle cells to a calcifying osteoblast-like phenotype. Diverse molecules such as BMP2, stearate, tumor necrosis factor α, high glucose, saturated fatty acids, parathyroid hormone, and C5a-C5aR1 have been shown to promote the osteogenic transition of VSMCs through ER stress induction (Liberman et al., 2011; Masuda et al., 2012; Masuda et al., 2013; Zhu et al., 2015; Shanahan and Furmanik, 2017; Shiozaki et al., 2018; Furmanik et al., 2021; Duang et al., 2022; Liu et al., 2023). Here we showed that the ER stress inhibitor 4-PBA prevents DPD-induced HAoSMCs and *ex vivo* aorta ring calcification (Figure 4), which observations prove that ER stress plays a key role in the pro-calcification effect of DPD.

ATF4 is an ER stress-induced pro-osteogenic transcriptional activator that has been identified as a central mediator of the ER stress-induced osteogenic transition of VSMCs and vascular calcification by several studies (Masuda et al., 2012; Masuda et al., 2013; Masuda et al., 2016; Furmanik and Shanahan, 2018). The most important proof of this notion is Masuda et al.'s study, which showed calcification attenuation in smooth muscle cell-specific ATF4 knock-out mice (Masuda et al., 2016). Our results also revealed that knockdown of ATF4 inhibits DPD-induced promotion of HAoSMC calcification (Figure 4). Therefore, the third key finding of this work is that ER stress and particularly ATF4 play a critical causative role in the pro-calcification effect of DPD.

DPD is a PHD inhibitor that initiates HIF signaling by stabilizing HIF alpha subunits of the HIF complex. Recent studies demonstrated that HIF activation, mediated either by hypoxia or PHD inhibition, promotes the phenotype switch of VSMCs into osteoblast-like cells under both normal and high phosphate conditions in a HIF-1α-dependent manner (Mokas et al., 2016; Balogh et al., 2019; Tóth et al., 2022; Csiki et al., 2023; Negri, 2023).

DPD induces both HIF-1α and ATF4 expressions in HAoSMCs. Growing evidence suggests bidirectional cooperation between HIF-1α and ATF4 in regulating diverse processes. For example, a single-allele deletion of HIF-1α is associated with lower CHOP expression and smaller infarct size in a mouse model of chronic intermittent hypoxia-mediated myocardial injury (Moulin et al., 2020). Here, using the siRNA approach to knockdown HIF-1α and ATF4, we found that HIF-1α is involved in DPD-induced upregulation of ATF4, but ATF4 does not control HIF-1α expression under these circumstances (Figure 5). Contradictory with this Chee et al. found that ATF4 regulates HIF-1α expression, but HIF-1α is not required for hypoxia-induced upregulation of ATF4 in pancreatic cancer cells (Chee et al., 2023). One explanation for this discrepancy could be that Chee et al. used 0.2% O_2_ to induce HIF-1α, while we used a prolyl hydroxylase inhibitor. Also, pancreatic cancer cells exist in a hypoxic environment while HAoSMCs live in a relatively well-oxygenated niche, which can lead to differences in their hypoxia responses. Nevertheless, further studies are needed to deepen our understanding of this phenomenon.

PHIs represent novel oral drug options for anemia management in patients with CKD. The use of PHIs is expected to rise, warranting further research to investigate the potential off-target effects of these drugs. In line with this notion, previously we have shown that DPD enhances vascular calcification in a mice model of CKD (Tóth et al., 2022), and here we described that DPD-induced activation of the PERK‐eIF2α‐ATF4‐CHOP axis of ER stress contributes to the pro-calcification effect of DPD. The limitation of our study is that we focused our work on DPD and have not tested the other PHIs; Roxadustat and Vadadustat. Another limitation of our work is that we performed our experiments exclusively in male C57BL/6 mice. Other mice strains and female mice should also be tested in the future. Nevertheless, to our knowledge, this is the first study showing that DPD induces ER stress *in vitro* and *in vivo*. ER stress is a key vascular calcification mechanism, therefore we strongly believe that this research can initiate further development to fine-tune PHIs for better and safer anemia management in CKD patients.

## Figures and Tables

**Table:** 

**AR**	alizarin red
**ATF4**	activating transcription factor 4
**ATF6**	activating transcription factor 6
**BMP2**	bone morphogenetic protein 2
**CHOP**	transcriptional factor C/EBP homologous protein
**CKD**	chronic kidney disease
**CKDD**	CKD treated with DPD
**Ctrl**	Control
**DMEM**	Dublecco’s modified eagle medium
**DMSO**	dimethyl sulphoxide
**DPBS**	Dulbecco’s phosphate-buffered saline
**DPD**	Daprodustat
**ECM**	extracellular matrix
**EDTA**	ethylenediamine-tetraacetic acid
**ER**	endoplasmic reticulum
**ESAs**	erythropoiesis-stimulating agents
**eIF2α**	eukaryotic initiation factor 2 alpha
**FBS**	fetal bovine serum
**FDA**	U.S. Food and Drug Administration
**Glut1**	glucose transporter 1
**GM**	growth medium
**GRP78**	glucose-regulated protein 78
**HAoSMC**	human aortic smooth muscle cell
**HIF**	hypoxia-inducible factor
**IRE1α**	inositol-requiring protein 1α
**MACE**	major cardiovascular event
**OCN**	Osteocalcin
**OD**	optical density
**OM**	osteogenic medium
**PBS**	phosphate-buffered saline
**P-eIF2α**	phospho-eIF2α
**PHD**	prolyl hydroxylase domain-containing
**PERK**	protein kinase RNA-like ER kinase
**Pi**	inorganic phosphate
**P-PERK**	phospho-PERK
**Runx2**	runt-related transcription factor 2
**4-PBA**	sodium-4-phenylbutyrate
**qPCR**	quantitative polymerase chain reaction
**Sox9**	SRY-box transcription factor 9
**VEGFA**	vascular endothelial growth factor A
**VSMCs**	vascular smooth muscle cells
**UPR**	unfolded protein response

## Data Availability

The data that support the findings of this study are available from the corresponding author upon reasonable request.
