# Supplementary material for: Activation of PERK/eIF2α/ATF4/CHOP branch of endoplasmic reticulum stress response and cooperation between HIF-1α and ATF4 promotes Daprodustat-induced vascular calcification
Source: Front Pharmacol. 2024 Jul 31;15:1399248. doi: 10.3389/fphar.2024.1399248 (PMC11322142; doi:10.3389/fphar.2024.1399248)
Supplement: Supplementary file 1 [file Table1.DOCX]

| **Cell line** | | |
| --- | --- | --- |
| **Name** | **Company** | **Catalog number** |
| Human Aortic Smooth Muscle Cells | Cell Applications Inc. (San Diego, CA, USA) | 354-05 |

| **Materials** | | | |
| --- | --- | --- | --- |
| **Name** | **Company** | **Catalog number** | **Concentration** |
| DMEM High Glucose | Sigma-Aldrich (St. Louis, MO, USA) | D6171 |  |
| Fetal Bovine Serum | Gibco (Grand Island, NY, USA) | 10270-106 |  |
| Sodium pyruvate | Sigma-Aldrich (St. Louis, MO, USA) | S8636 |  |
| L-Glutamine | Sigma-Aldrich (St. Louis, MO, USA) | G7513 |  |
| Antibiotic antimycotic solution | Sigma-Aldrich (St. Louis, MO, USA) | A5955 |  |
| NaH2PO4 | Sigma-Aldrich (St. Louis, MO, USA) | S5011 |  |
| Na2HPO4 | Sigma-Aldrich (St. Louis, MO, USA | S5136 |  |
| Daprodustat (DPD) | MedChemExpress  (Monmouth Junction, NJ, USA) | HY-17608 | 1-100 µmol/L |
| Methly cellulose | Sigma-Aldrich (St. Louis, MO, USA) | M0512 | 1% |
| DPBS | Gibco (Grand Island, NY, USA) | 14190-144 |  |
| Sodium-4-phenyl-butyrate (4-PBA) | Cayman Chemicals  (Ann Arbor, Michigan, USA) | 11323 | 250 µmol/L |
| DMSO | Sigma-Aldrich (St. Louis, MO, USA) | D2438 |  |
| Fungizone (Amphotericin B) | Merck  (Darmstadt, Germany) | 171375 | 2.5 µg/ml |
| Paraformaldehyde | Merck | 16005 |  |
| Alizarin Red S | Sigma-Aldrich (St. Louis, MO, USA) | A5533 | 2% |
| Hexadecyl-pyridinium chloride | Sigma-Aldrich (St. Louis, MO, USA) | C9002 | 100 mmol/L |
| HCl | Sigma-Aldrich (St. Louis, MO, USA) | 30721 | 0.6 mol/L |
| NaOH | Sigma-Aldrich (St. Louis, MO, USA) | S8045 | 0.1 mol/L |
| Sodium Dodecyl Sulfate | Sigma-Aldrich (St. Louis, MO, USA) | 11667289001 | 0.1% |
| EDTA | Sigma-Aldrich (St. Louis, MO, USA) | E6758 |  |
| Tri reagent | Sigma-Aldrich (St. Louis, MO, USA) | T9424 |  |
| OsteoSense 680EX | PerkinElmer (Waltham, MA, USA) | NEV10020EX | 2 nmol |
| 0.2% adenine and 0.7% phosphate diet | Ssniff (Soest, Germany) | S8106-S075 |  |
| 0.2% adenine and 1.8% phosphate diet | Ssniff (Soest, Germany) | S8893-S006 |  |
| Nitrocellulose membrane | Amersham Protran,  (GE Healthcare, Chicago, IL, USA) | 10600002 |  |
| Isoflurane | Baxter (Deerfield, IL, USA) | 6DG9621 |  |

| **Antibodies** | | | |
| --- | --- | --- | --- |
| **Name** | **Company** | **Catalog number** | **Concentration** |
| anti-Glut1 | GeneTex (Irvine, CA, USA) | GTX1309 | 1:500 (0.5 µg/ml) |
| anti-HIF-1α | GeneTex (Irvine, CA, USA) | GTX127309 | 1:1000 (0.5 µg/ml) |
| anti-ATF4 | Cell Signaling Tech (Danvers, MA, USA) | #11815 | 1:1000 (0.3 µg/ml) |
| anti-PERK | Cell Signaling Tech (Danvers, MA, USA) | #3192 | 1:1000 (0.5 µg/ml) |
| anti-phospho PERK | Invitrogen (Carlsbad, CA, USA) | PA5-4029 | 1:1000 (5 μg/ml) |
| anti-eIF2α | Cell Signaling Tech (Danvers, MA, USA) | #9722 | 1:800 (0.06 μg/ml) |
| anti-phospho eIF2α | Cell Signaling Tech (Danvers, MA, USA) | #9721 | 1:800 (0.6 μg/ml) |
| anti-CHOP | Novusbio (Centennial, CO, USA) | NB600-1335 | 1:1000 (4 μg/ml) |
| Rabbit IgG HRP | Amersham, GE Healthcare (Chicago, IL, USA) | NA-934 | 1:10 000  (0.5 μg/ml) |
| Mouse IgG HRP | Amersham, GE Healthcare (Chicago, IL, USA) | NA-931 | 1:10 000  (0.5 μg/ml) |
| anti-β-actin | Santa Cruz Biotechnology Inc. (Dallas, TX, USA) | sc-47778 | 1:4000 (0.5 μg/ml) |

| **Kits** | | |
| --- | --- | --- |
| **Name** | **Company** | **Catalog number** |
| Clarity Western ECL | BioRad (Hercules, CA, USA) | 170-5061 |
| QuantiChrome Calcium Assay Kit | Gentaur (Kampenhout, Belgium) | DICA-500 |
| BCA protein assay kit | Pierce Biotechnology (Rockford, IL, USA) | 23225 |
| iTaqTM Universal SYBR® Green Supermix | Bio-Rad (Hercules, CA, USA) | 1725124 |
| OCN Enzyme-linked immunosorbent assay | DuoSet ELISA (R&D, Minneapolis, MN, USA) | DY1419-05 |
| High Capacity cDNA Reverse Transcription kit | Applied Biosystems (Waltham, MA, USA) | 4368813 |
| Lipofectamine RNAiMAX transfection reagent | Invitrogen (Carlsbad, CA, USA) | 13778-150 |

| **RNA silencers** | | | |
| --- | --- | --- | --- |
| **Name** | **Company** | **Catalog number** | **Concentration** |
| Silencer Select Negative Control | Invitrogen  (Carlsbad, CA, USA) | 4390843 | 10 µmol/L |
| siRNA for ATF4 | Invitrogen  (Carlsbad, CA, USA) | AM16708, ID: s1702 | 10 µmol/L |
| siRNA for HIF1 | Invitrogen  (Carlsbad, CA, USA) | AM16708, ID: 106498 | 10 µmol/L |

| **Primers** | | | | |
| --- | --- | --- | --- | --- |
| **Name** | **Sequence** | **Company** | **Concentration** | |
| HPRT FW | 5’-TCCTCCTCAGACCGCTTTT-3’ | Sigma-Aldrich | | 10 µmol/L |
| HPRT Rev | 5’-CCTGGTTCATCATCGCTAATC-3’ | Sigma-Aldrich | | 10 µmol/L |
| GLUT1 FW | 5’-GGCCATCTTTTCTGTTGGGG-3’ | Sigma-Aldrich | | 10 µmol/L |
| GLUT1 Rev | 5’-CCAGCAGGTTCATCATCAGC-3’ | Sigma-Aldrich | | 10 µmol/L |
| VEGF FW | 5’-CTACCTCCACCATGCCAAGT-3’ | Sigma-Aldrich | | 10 µmol/L |
| VEGF Rev | 5’-GATAGACATCCATGAACTTCACCA-3’ | Sigma-Aldrich | | 10 µmol/L |
| ACTB FW | 5’-GAGGTATCCTGACCCTGAAGTA-3’ | Sigma-Aldrich | | 10 µmol/L |
| ACTB Rev | 5’-TCTACAATGAGCTGCGTGTG-3’ | Sigma-Aldrich | | 10 µmol/L |
| ATF4 FW | 5’-AATGGCCGGCTATGGATGAT-3’ | Sigma-Aldrich | | 10 µmol/L |
| ATF4 Rev | 5’-CAATCTGTCCCGGAAAAGGC-3’ | Sigma-Aldrich | | 10 µmol/L |
| CHOP FW | 5’-AAGCCTGGTATGAGGATCTGC-3’ | Sigma-Aldrich | | 10 µmol/L |
| CHOP Rev | 5’-TTCCTGGGGATGAGATATAGGTG-3’ | Sigma-Aldrich | | 10 µmol/L |
| Grp78 FW | 5’-TCGGGCCAAATTTTGAAGAGC-3’ | Sigma-Aldrich | | 10 µmol/L |
| Grp78 Rev | 5’-CAACACTTTCTGGACAGGCT-3’ | Sigma-Aldrich | | 10 µmol/L |
| Runx2 FW | 5’-GCATCCTATCAGTTCCCAATG-3’ | Sigma-Aldrich | | 10 µmol/L |
| Runx2 Rev | 5’-GAGGTGGTGGTGCATGGT-3’ | Sigma-Aldrich | | 10 µmol/L |
| Sox9 FW | 5’-GCTCTACTCCACCTTCACTTAC-3’ | Sigma-Aldrich | | 10 µmol/L |
| Sox9 Rev | 5’-TGTGTGTAGACTGGTTGTTCC-3’ | Sigma-Aldrich | | 10 µmol/L |
| BMP-2 FW | 5’-CGGACTGCGGTCTCCTAA-3’ | Sigma-Aldrich | | 10 µmol/L |
| BMP-2 Rev | 5’-GGGGAAGCAGCAACACTAGA-3’ | Sigma-Aldrich | | 10 µmol/L |
| MSX-2 FW | 5’-AGGAGCCCGGCAGATACT-3’ | Sigma-Aldrich | | 10 µmol/L |
| MSX-2 Rev | 5’-GTTTCCTCAGGGTGCAGGT-3’ | Sigma-Aldrich | | 10 µmol/L |

| **Instruments** | |
| --- | --- |
| **Name** | **Company** |
| C-Digit Blot Scanner | LI-COR Biosciences (Lincoln, NE, USA) |
| BioTek ELISA Reader 800TS | Agilent (Santa Clara, CA, USA) |
| IVIS Spectrum In Vivo Imaging System | PerkinElmer (Waltham, MA, USA) |
| CFX96 Real-Time System | Bio-Rad Laboratories (Hercules, CA, USA) |
| Cobas c502 | Roche Diagnostics (Mannheim, Germany) |
